# Malnutrition-related mortality trends in older adults in the United States from 1999 to 2020

**DOI:** 10.1186/s12916-023-03143-8

**Published:** 2023-11-07

**Authors:** Naydeen Mostafa, Ahmed Sayed, Omar Rashad, Omar Baqal

**Affiliations:** 1 Faculty of Medicine, Ain Shams University, Cairo, Egypt; 2 Department of Internal Medicine, Mayo Clinic Arizona, Phoenix, AZ USA

**Keywords:** Malnutrition, Geriatrics, Mortality trends, Public health

## Abstract

**Background:**

Malnutrition mortality in older adults is underrepresented in scientific literature. This obscures any recent changes and hinders needed social change. This study aims to assess malnutrition mortality trends in older adults (≥ 65 years old) from 1999 to 2020 in the United States (U.S.).

**Methods:**

Mortality data from the Centers for Disease Control and Prevention’s (CDC) Wide-Ranging Online Data for Epidemiology Research (WONDER) database were extracted. The ICD-10 Codes E40 – E46 were used to identify malnutrition deaths. Crude mortality rates (CMR) and age-adjusted mortality rates (AAMR) were extracted by gender, age, race, census region, and urban–rural classification. Joinpoint regression analysis was used to calculate annual percentage changes (APC) of AAMR by the permutation test and the parametric method was used to calculate 95% confidence intervals. Average Annual Percentage Changes (AAPC) were calculated as the weighted average of APCs.

**Results:**

Between 1999 and 2020, 93,244 older adults died from malnutrition. Malnutrition AAMR increased from 10.7 per 100,000 in 1999 to 25.0 per 100,000 in 2020. The mortality trend declined from 1999 to 2006 (APC = –8.8; 95% CI: –10.0, –7.5), plateaued till 2013, then began to rise from 2013 to 2020 with an APC of 22.4 (95% CI: 21.3, 23.5) and an overall AAPC of 3.9 (95% CI: 3.1, 4.7). Persons ≥ 85 years of age, females, Non-Hispanic Whites, residents of the West region of the U.S., and urban areas had the highest AAPCs in their respective groups.

**Conclusion:**

Despite some initial decrements in malnutrition mortality among older adults in the U.S., the uptrend from 2013 to 2020 nullified all established progress. The end result is that malnutrition mortality rates represent a historical high. The burden of the mortality uptrends disproportionately affected certain demographics, namely persons ≥ 85 years of age, females, Non-Hispanic Whites, those living in the West region of the U.S., and urban areas. Effective interventions are strongly needed. Such interventions should aim to ensure food security and early detection and remedy of malnutrition among older adults through stronger government-funded programs and social support systems, increased funding for nursing homes, and more cohesive patient-centered medical care.

## Background

Malnutrition is a complex multi-factorial condition defined as a decrease in body fat and/or muscle mass due to diminished intake or absorption of nutrients [1]. Malnutrition negatively impacts everyday activity and is associated with poor quality of life and physical deterioration [2, 3]. Additionally, malnutrition is linked to higher rates of morbidity and mortality [4]. On a population level, the frequent and prolonged hospital admissions associated with malnutrition exert a tremendous financial burden on the healthcare system [5, 6].

Malnutrition can impact people of all ages. However, older adults (≥ 65 years old) can be particularly susceptible due to a combination of risk factors [3]. Aging causes physiological decline ranging from fading taste and smell to reduced appetite and impaired gastrointestinal absorption [3]. Age-associated loss of teeth can further exacerbate poor nutritional status among older adults [1]. They are also more likely to develop depression and social loneliness, which are independent risk factors for malnutrition [7, 8].

Estimates show that around 25% of older adults are malnourished or at risk of malnutrition with an expected rise as the population continues to age [9]. By 2030, older adults are projected to comprise 20% of the United States (U.S.) population [10] with the risk of malnutrition predicted to increase alongside the rise in life expectancy [11]. The scarcity of recent literature on malnutrition mortality trends in the older adult population obscures the magnitude of this public health problem. Accordingly, we sought to assess the trends of malnutrition mortality in older adults from 1999 to 2020 in the U.S. to provide policymakers with data on the scope of this public health issue. To the best of our knowledge, this is the first study to analyze malnutrition mortality trends among older adults in the U.S.

## Methods

### Data extraction

This study used data produced by the National Center for Health Statistics (NCHS) available through the Centers for Disease Control and Prevention Wide-Ranging On-Line Data for Epidemiologic Research (CDC WONDER) database [12]. Data are updated annually based on U.S. residents’ death certificates, which contain an underlying cause of death and demographic data. Malnutrition as the underlying cause of death was identified using the International Classification of Diseases, 10th Revision (ICD-10 code: E40-E46). From 1999 to 2020, crude mortality rate (CMR) and age-adjusted mortality rate per 100,000 (AAMR) were extracted with their respective confidence intervals (CI) and standard errors (SE). AAMR was based on the 2000 standard American population [13]. Mortality rates were then extracted by gender, race, age group, place of death, census region, and urban–rural classification. For the urban–rural classification, the 2013 NCHS Urban–Rural Classification Scheme was used [14] which divides urban counties into large central metropolitan (≥ 1,000,000 population), large fringe metropolitan (≥ 1,000,000 population, but does not qualify as large central metropolitan), medium metropolitan (250,000 – 9999,999 population), and small metropolitan (< 250,000 population), and divides rural counties into micropolitan (10,000 – 49,000 urban cluster population) and noncore (< 10,000 urban cluster population). We only included data for individuals aged 65 years and above.

### Statistical analysis

To analyze mortality trends from 1999 to 2020, Joinpoint Regression Program V 5.0.0.0, provided by the National Cancer Institute (NCI), was used [15]. This program identifies changes in AAMR to compute annual percentage change (APC) by fitting a log-linear regression model where temporal variation occurred. Average Annual Percentage Changes (AAPC) over 1999–2020 were calculated as the weighted average of APCs. The minimum and maximum number of joinpoints used was according to the guidelines [16] which were 0 to 4. The final selected model by Joinpoint was used. The APC/AAPC/Tau confidence interval was calculated using the parametric method. The permutation test was run with 4499 permutations. A *P* value of < 0.05 denoted statistical significance. Institutional review board approval was not required as all data used are in the public domain and de-identified.

## Results

There were 93,244 recorded malnutrition deaths in older adults from 1999 to 2020 in the U.S. Of these deaths, 34.3% took place inside medical facilities, 30.2% inside nursing homes/long-term care facilities, and 25.6% inside the descendant’s home. Overall, AAMR was 10 per 100,000 (95% CI: 10, 10.1). Mortality rates were highest in females (AAMR = 10.3; 95% CI: 10.2, 10.3), Non-Hispanic Blacks (AAMR = 14.6; 95% CI: 14.3, 14.9), persons aged ≥ 85 years (CMR = 44.8; 95% CI: 44.4, 45.2), residents of the South region (AAMR = 12.4; 95% CI: 12.3, 12.5), and small metropolitan counties (AAMR = 11.9; 95% CI: 11.7, 12.2) **(**Table 1).

**Table 1:** Demographic composition of the standardized US population in 2000 and malnutrition deaths occurring from 1999–2020

Variable	Standard U.S. population in 2000 n (%)	Malnutrition deaths n (%)	Age-adjusted mortality rate per 100,000 (95% CI)
Older adults	34,991,753 (100)	93,244 (100)	10 (10.0, 10.1)
Gender			
Female	20,582,128 (58.8)	60,104 (64.5)	10.3 (10.2, 10.3)
Male	14,409,625 (41.2)	33,140 (35.5)	9.5 (9.4, 9.6)
Race			
White	29,430,960 (84.1)	75,171 (80.6)	9.8 (9.7, 9.9)
Black	2,831,708 (8.1)	10,629 (11.4)	14.6 (14.3, 14.9)
Asian /Pacific Islander	855,554 (2.4)	1,928 (2.1)	6.4 (6.1, 6.7)
American Indian/ Alaska Native	139,937 (0.4)	596 (0.6)	15.7 (14.4,17.0)
Hispanic/Latino	1,773,591 (5.1)	4,721 (5.1)	8.4 (8.1, 8.6)
Census region			
Northeast	7,372,282 (21.1)	11,433 (12.3)	5.9 (5.8, 6.0)
Midwest	8,259,075 (23.6)	23,183 (24.9)	10.7 (10.6, 10.9)
South	12,438,267 (35.5)	40,597 (43.5)	12.4 (12.3, 12.5)
West	6,922,129 (19.8)	18,031 (19.3)	9.2 (9.1, 9.3)
Age group			
65 – 74	18,390,986 (52.6)	12,606 (13.5)	2.5 (2.4, 2.5)
75 – 84	12,361,180 (35.3)	27,079 (29.0)	9.1 (9.0, 9.2)
≥ 85	4,239,587 (12.1)	53,559 (57.4)	44.8 (44.4, 45.2)
Urban–rural areas			
Large Central Metropolitan	9,721,383 (27.8)	22,482 (24.1)	8.8 (8.7, 9.0)
Large Fringe Metropolitan	7,776,987 (22.2)	19,618 (21.0)	8.9 (8.9, 9.0)
Medium Metropolitan	7,342,976 (21.0)	21,468 (23.0)	10.8 (10.7, 11.0)
Small Metropolitan	3,469,622 (9.1)	11,035 (11.8)	11.9 (11.7, 12.2)
Micropolitan	3,661,442 (10.5)	10,769 (11.5)	11.6 (11.4, 11.8)
Noncore	3,019,343 (8.6)	7,872 (8.4)	10.6 (10.4, 10.8)

### Overall trends

**Fig. 1:**
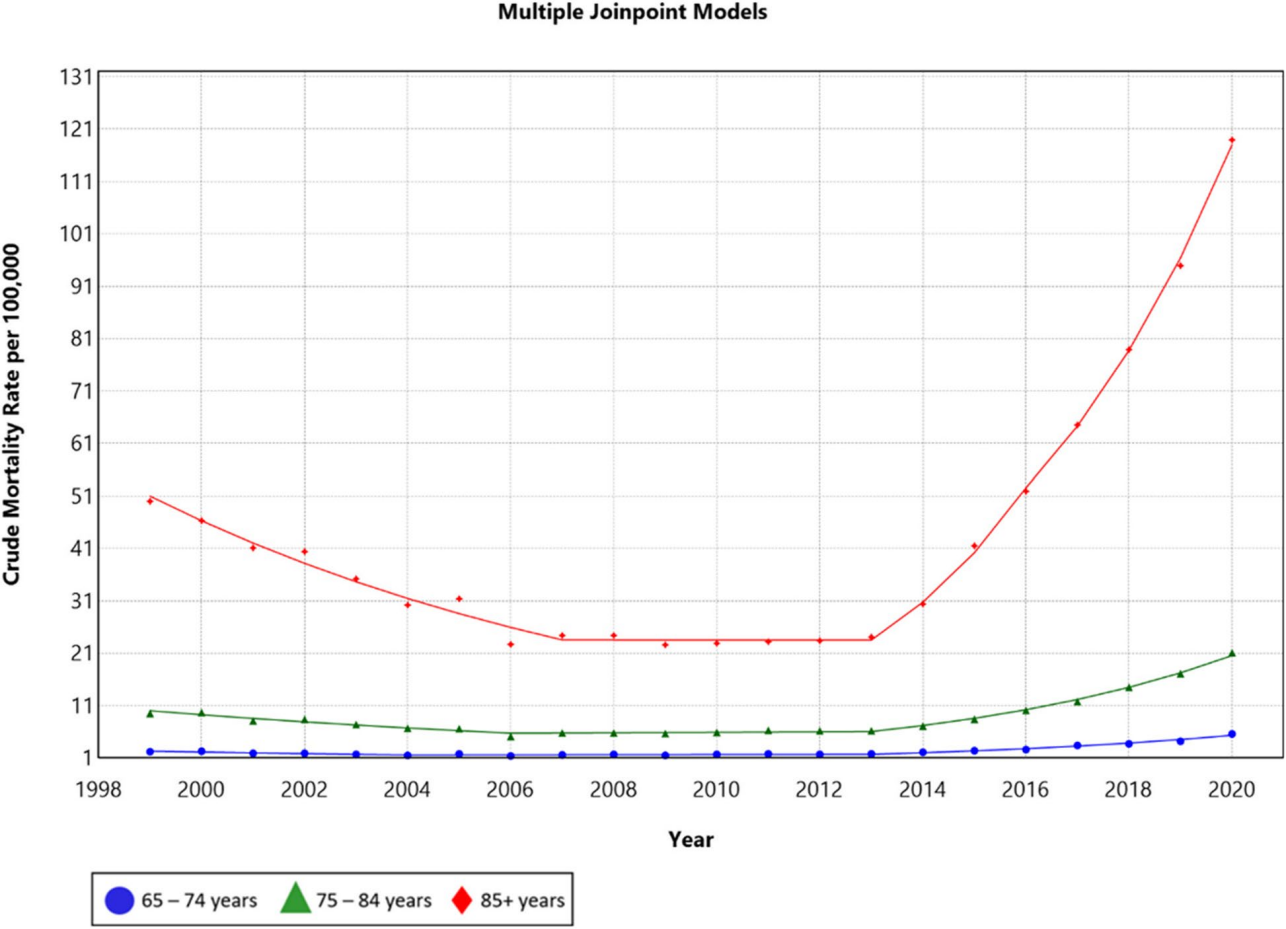
Trends in age-adjusted mortality rate (per 100,000) for malnutrition among U.S. older adults from 1999 to 2020 stratified by age group. The Joinpoint regression model identified three time segments for the 65 – 74 and the 75 – 84 age groups and four time segments for the ≥ 85 age group. For the 65 – 74 age group, 1999 to 2004 APC: –7.1 (95 CI%: –12.6, –1.4), 2004 to 2013 APC: 0.80 (95% CI: –2.6, 4.3), 2013 to 2020 APC: 17.6 (95% CI: 15.1, 20.2). For the 75 – 84 age group, 1999 to 2006 APC: –7.6 (95% CI: –8.8, –6.4), 2006 to 2013 APC: 0.7 (95% CI: –1.1, 2.5), 2013 to 2020 APC: 19.1 (95% CI: 18.0, 20.2). For the ≥ 85 age group, 1999 to 2007 APC: –9.2 (95% CI: –10.8, –7.6), 2007 to 2013 APC: 0.0 (95% CI: –3.9, 4.0), 2013 to 2016 APC: 30.7 (95% CI: 13.8, 50.1), 2016 to 2020 APC: 22.4 (95% CI: 19.2, 25.8)

From 1999 to 2020, AAMR increased from 10.7 to 25.0 (AAPC = 3.9; 95% CI: 3.1, 4.7). There was a significant gradual decrease from 1999 to 2006 (APC = –8.8; 95% CI: –10.0, –7.5). This was followed by a near-plateau from 2006 to 2013 (APC = 0.5, 95% CI: –1.3, 2.3), which was followed by a significant increase from 2013 to 2020 (APC = 22.4; 95% CI: 21.3, 23.5) (Fig. 1).

### Trends by age

Through our study period, older ages had higher AAMRs, with the 2020 AAMR being 5.6 (95% CI: 5.3, 5.8) in the 65 – 74 age group, 21.1 (95% CI: 20.4, 21.8) in the 75 – 84 age group, and 118.9 (95% CI: 116.3, 121.5) in the ≥ 85 age group. On the other hand, the age groups with the highest AAPC were the ≥ 85 age group (AAPC = 4.1; 95% CI: 1.9, 6.4) and the 65 – 74 age group (AAPC = 4.1; 95% CI: 2.0, 6.2), followed by the 75 – 84 years age group (AAPC = 3.5; 95% CI: 2.7, 4.2) (Fig. 1).

### Trends by gender

**Fig. 2:**
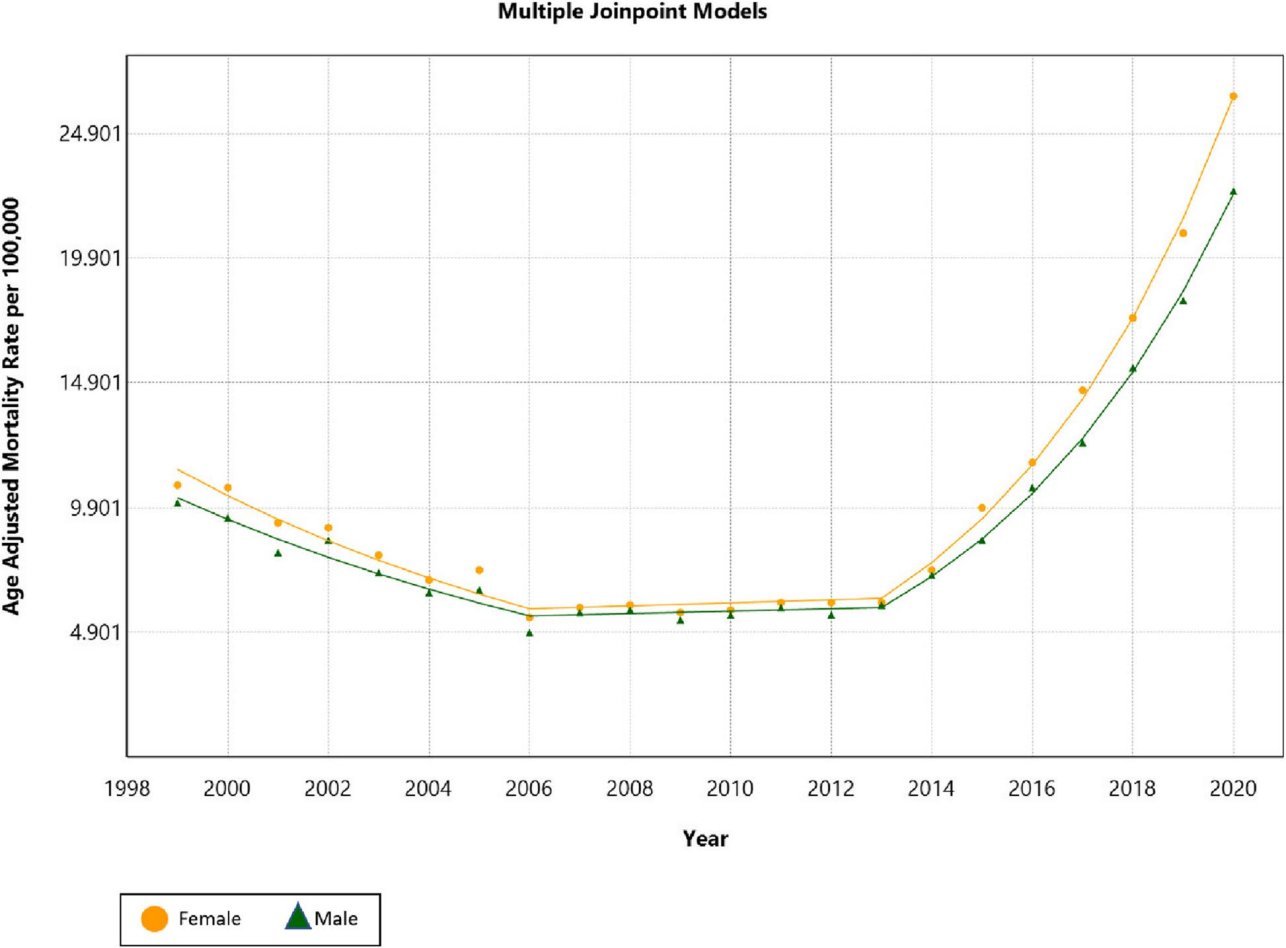
Trends in age-adjusted mortality rate (per 100,000) for malnutrition among U.S. older adults from 1999 to 2020 stratified by gender. The Joinpoint regression model identified three time segments for both genders. For females, 1999 to 2006 APC: –9.1 (95% CI: –10.5, –7.7), 2006 to 2013 APC: 1.0 (95% CI: –1.8, 3.8), 2013 to 2020 APC: 22.8 (95% CI: 21.4, 24.2). For males, 1999 to 2006 APC: –8.4 (95% CI: –9.8, –7.1), 2006 to 2013 APC: 0.8 (95% CI: –1.3, 3.0), 2013 to 2020 APC: 21.1 (95% CI: 19.9, 22.2)

Similar to the overall population, both males and females benefited from an initial decline in AAMR, followed by a succeeding increase from 2013 to 2020. The annual AAMR was consistently higher in females with an overall AAMR of 10.3 (95% CI: 10.2, 10.3) compared to 9.5 (95% CI: 9.4, 9.6) in males. Furthermore, Females had higher AAPC (AAPC = 4.1; 95% CI: 3, 5.1 versus AAPC = 3.8; 95% CI: 2.9, 4.7) from 1999 to 2020 (Fig. 2).

### Trends by race/ethnicity

**Fig. 3:**
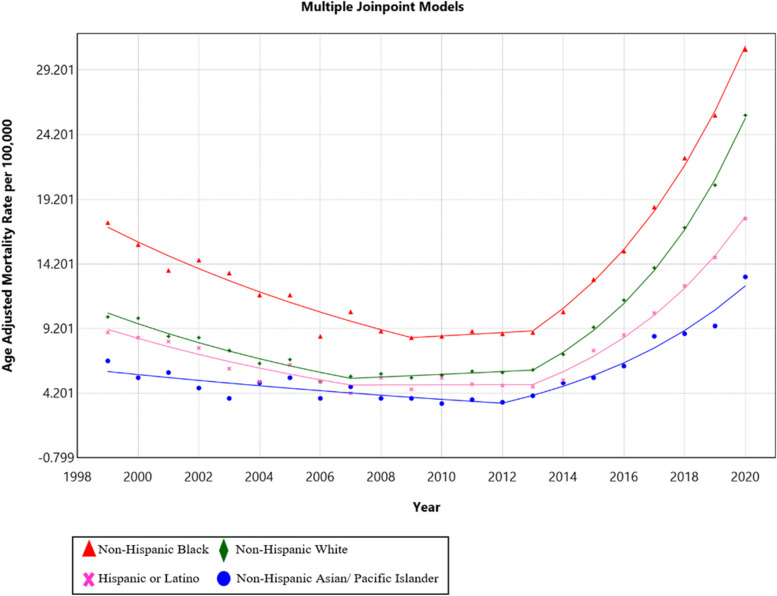
Trends in age-adjusted mortality rate (per 100,000) for malnutrition among U.S. older adults from 1999 to 2020 stratified by race. The Joinpoint regression model identified three time segments for Non-Hispanic Blacks, Non-Hispanic Whites, and Hispanics, and two time segments for Non-Hispanic Asian/Pacific Islanders. For Non-Hispanic Blacks, 1999 to 2009 APC: –6.7 (95% CI: –8.0, –5.4), 2009 to 2013 APC: 1.5 (95% CI: –7.7, 11.6), 2013 to 2020 APC: 19.3 (95% CI: 17.4, 21.2). For Non-Hispanic Whites, 1999 to 2007 APC: –8.0 (95% CI: –9.6, 6.3), 2007 to 2013 APC: 1.9 (95% CI: –1.5, 5.4), 2013 to 2020 APC: 23.0 (95% CI: 21.1, 24.9). For Hispanics, 1999 to 2007 APC: –7.6 (95% CI: –10.3, –4.9), 2007 to 2013 APC: 0.1 (95% CI: –5.3, –5.9), 2013 to 2020 APC: 20.4 (95% CI: 18.0, 22.8). For Non-Hispanic Asian/Pacific Islanders, 1999 to 2012 APC: –4.1 (95% CI: –6.2, –1.9), 2012 to 2020 APC: 17.6 (95% CI: 14.7, 20.5)

While AAMR was highest in Non-Hispanic Blacks (AAMR = 14.6; 95% CI: 14.3, 14.9), Non-Hispanic Whites had the highest AAPC (AAPC = 4.4; 95% CI: 3.1, 5.6). Non-Hispanic Asians had an AAPC of 3.7 (95% CI: 2.1, 5.3). Hispanics had an AAPC of 3.2 (95% CI: 1.3, 5.2). Lastly, Non-Hispanic Blacks had an AAPC of 2.9 (95% CI: 1.0, 4.8). A detailed summary of the results is provided in Fig. 3.

### Trends by region and county

**Fig. 4:**
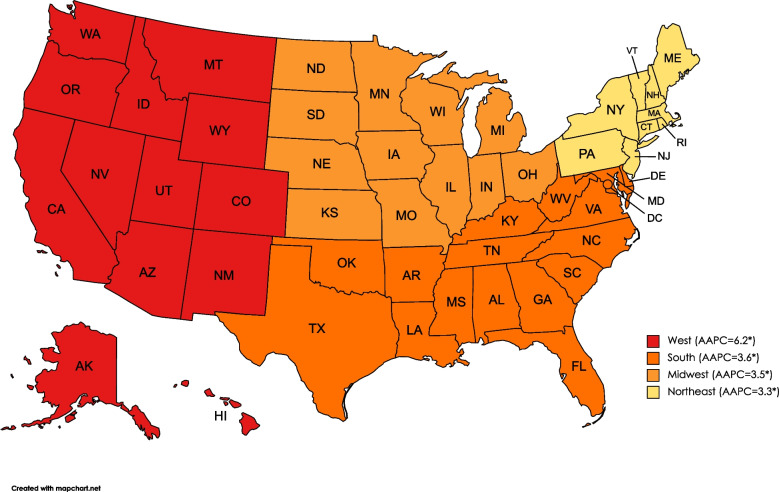
Average annual percentage change of age-adjusted mortality rate (per 100,000) for malnutrition among U.S. older adults from 1999 to 2020 in U.S. census regions. * = significant at *p* < 0.05; confidence interval does not include zero

Trends for regions and counties mirrored the trends for the overall population, with an initial decline in malnutrition AAMR which was subsequently nullified by a strong uptrend. The rate of increase differed by different regions and counties. Overall, the West had the highest AAPC (AAPC = 6.2; 95% CI: 5.1, 7.4) as its AAMR increased from 7.6 in 1999 to 27.4 in 2020, while Northeast had the lowest AAPC over the study period (AAPC = 3.3; 95% CI: 1.2, 5.4) (Fig. 4).

**Fig. 5:**
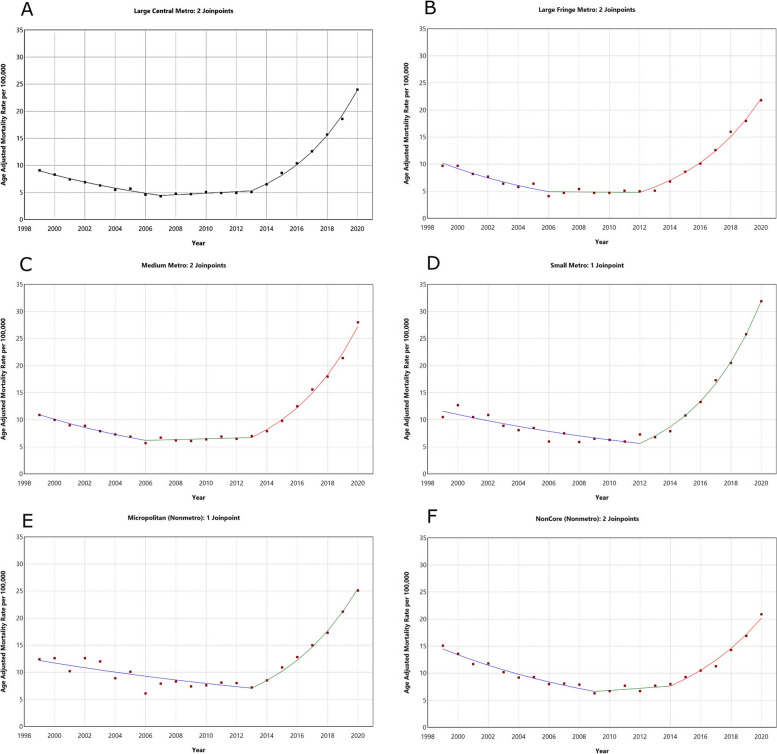
Trends in age-adjusted mortality rate (per 100,000) for malnutrition among U.S. older adults from 1999 to 2020 stratified by urban–rural 2013 classification. The Joinpoint regression model identified three time segments for (**A**), (**B**), (**C**), and (**F**), and two time segments for (**E**) and (**D**). **A** 1999 to 2007 APC: –8.4 (95% CI: –9.6, –7.2), 2007 to 2013 APC: 3.0 (95% CI: 0.0, 6.1), 2013 to 2020 APC: 24.0 (95% CI: 22.7, 25.3). **B** 1999 to 2006 APC: –9.9 (95% CI: –12.6, –7.1), 2006 to 2013 APC: 0.5 (95% CI: –5.0, 4.2), 2013 to 2020 APC: 21.1 (95% CI: 19.3, 22.9). **C** 1999 to 2006 APC: –7.8 (95% CI: –9.5, –6.0), 2006 to 2013 APC: 1.2 (95% CI: –1.6, 4.0), 2013 to 2020 APC: 22.1 (95% CI: 20.7, 23.6). **D** 1999 to 2012 APC: –5.4 (95% CI: –6.7, –4.1), 2012 to 2020 APC: 24.2 (95% CI: 21.8, 26.7). **E** 1999 to 2013 APC: –3.8 (95% CI: –5.3, –2.3), 2012 to 2023 APC: 20.1 (95% CI: 16.1, 24.2). **F** 1999 to 2009 APC: –7.5 (95% CI: –8.8, –6.2), 2009 to 2014 APC: 2.8 (95% CI: –4.2, 10.4), 2014 to 2020 APC: 17.6 (95% CI: 14.8, 20.4)

Urban counties had an AAPC of 4.4 (95% CI: 3.5, 5.2) while rural counties had an AAPC of 2.5 (95% CI: 1.5, 3.6). Further subgroup analysis showed that small metropolitan counties had the highest AAPC. (AAPC = 4.9; 95% CI: 3.8, 6.1). Noncore counties (most rural) had the lowest AAPC (AAPC = 1.6; 95% CI: –0.2, 3.4) and the change was not statistically significant. The counties with the lowest significant AAPC were micropolitan (AAPC = 3.6, 95% CI: 2.1, 5.1). A summary of the subgroup analysis is provided in Fig. 5.

## Discussion

Our study highlights a worrying trend in malnutrition-related deaths among older adults. Although an initial period of decline in malnutrition mortality was seen from 1999 to 2006, this was not long-lasting. It was followed by a plateau between 2006 and 2013 and an eventual uptrend from 2013 to 2020, at a rate sharper than the initial rate of decline. The end result is that malnutrition mortality rates among older adults in the U.S. are at a historical high.

The increase in mortality rates is likely attributable to a confluence of risk factors for malnutrition rather than a singular culprit. Further, we suspect the changing mortality trends between 1999 and 2020 were due to the delicate balance between risk factors and protective factors for malnutrition, which at different points in time, succeeded in outpowering the other.

Because economic prosperity has long been established as a crucial determinant of population health [17, 18], the potential role of economic factors in driving the trend is critical to note. At the beginning of the period of trend decline, the U.S. was still experiencing one of its longest economic expansions in history, coming out of the 1990s boom [19]. A mild eight-month recession in 2001, was followed by more economic growth for several years [20]. Furthermore, the U.S. at this point was spending more on healthcare than any other developed nation in the world, becoming a world leader in health technology [21] and health research [22]. The systemic factors supporting health in this period decreased all-cause AAMR in U.S. older adults from 5,220 per 100,000 in 1999 to 4640 per 100,000 in 2006 [12].

The economic stressors that followed during the Great Recession may have placed tremendous financial and psychological stress on older adults, particularly the economically disadvantaged. This destabilization of protective factors for malnutrition was likely responsible for the plateau in the mortality trend from 2006 to 2013.

After years of progressively waning, protective factors could not prevent the 2013 uptrend in mortality. While the U.S. continued its astronomical spending on healthcare, the value of healthcare spending in the older adult population has been decreasing over time [23]. Upon closer scrutiny, it became clear the high U.S. healthcare spending could have potentially started to drive up the mortality trend. Two of the reasons responsible for the high cost of U.S. healthcare are care fragmentation (the dispersion of a patient’s healthcare across systems and providers) [24] and unregulated fee-for-service. Care fragmentation can cause poor communication between healthcare providers with sometimes contradicting instructions being given to patients, while unregulated fee-for-service can lead to unneeded procedures, with risks of secondary complications [23]. A recent study on the U.S. medicine floors found that three-fourths of patients with malnutrition had at least a single gap of care, the most common of which involved discharge diet instructions, procedures, and inadequate communication, hindering interventions [25]. In line with the counterproductive nature of care fragmentation, screening of older adults for malnutrition is still not routinely performed – despite malnutrition being preventable with screening and appropriate interventions – because there is doubt about which healthcare professional should perform the screening [9].

Moreover, the high spending on healthcare could be driving resources away from public health problems in need of funding and contributing to malnutrition. For example, despite several studies showing the training of nursing home staff on diet quality improvement and feeding assistance care leads to improvement of malnutrition indices of residents [26, 27], funding for such quality improvement projects is still deficient [28].

More broadly, the fall of protective factors for geriatric health is reflected in other indices: all-cause mortality in older adults only declined from an AAMR of 4267 per 100,000 in 2013 to an AAMR of 4074 per 100,000 in 2019. The initial decline in mortality rate from 1999 to 2006 (decline of ~ 600) far outpaced the decline seen from 2013 to 2019 (decline of ~ 200) [12]. Additionally, specific causes of death, aside from malnutrition, in older adults were also on the rise in the same timeframe as malnutrition [29, 30] and U.S. life expectancy, despite decades of persistent increases, decreased for three consecutive years after 2014 [31]. This reinforces the argument that protective factors, which were once able to decrease malnutrition mortality, were systemic in nature.

Further contributing to the recent uptrend in malnutrition mortality are the rising risk factors for malnutrition. Among the number of potential culprits is a growing burden of comorbidities. For instance, depression is an established risk factor for malnutrition [32], due to its ability to decrease appetite and food intake [33]. In the rural setting, older adults with depression were more than three times more likely to be malnourished than their non-depressed counterparts [34]. Similar to the malnutrition mortality uptrend, the estimated prevalence of past-year major depressive disorder in older adults increased by a startling 60% from 2010/11 to 2018/19 [35].

Diseases, outside the realm of mental health disorders, have also been shown to increase the risk of malnutrition [36]. Considering more than 80% of older adults in the U.S. have at least one chronic disease [37], this may be an important additional contributor to our findings given the significant burden of malnutrition among these patients [38–40]. Moreover, trends for chronic diseases showed an overall increase in the same timeframe as the start of the malnutrition mortality uptrend [41].

The growing utilization of nursing homes, whose utilization has more than doubled between 2000 and 2013 [42] may also be an important aspect to consider [43]. A study collecting and analyzing data from seven nursing home rehabilitation wards found that more than one out of three patients were moderately to severely malnourished [44]. It is difficult to assert causality from nursing homes as they typically house vulnerable older adults [45] who are inherently predisposed to malnutrition. Nevertheless, the adoption of effective interventions that can detect early signs of malnutrition is an important step in decreasing its prevalence in nursing homes.

Other maladies that defy biomedical labels but which are nevertheless crucial in influencing geriatric health should also be considered. A previous study identified social isolation and subjective loneliness, two growing public health concerns [46], as independent risk factors for malnutrition in older adults [47]. A further pivotal risk factor for malnutrition is food insecurity [48, 49], a state of limited access to food due to a lack of financial resources [50]. A cross-sectional study on community-dwelling older adults reported that any category of food insecurity, even if mild, increased the risk of malnutrition relative to food-secure older adults [51]. In line with the malnutrition mortality trend, analysis of the National Health and Nutrition Examination Surveys (NHANES) showed that food insecurity increased among older adults by more than two-fold from 2007 to 2016 (increasing from 5.5% to 12.4% respectively) [52]. This increase in food insecurity was most notable among those with lower incomes. Indeed, national reports show that 26.4% of U.S. older adults below the poverty line suffer from food insecurity compared to only 2.7% of older adults with incomes greater than twice the poverty line [53]. A county-level analysis combining data from the American Community Survey, CDC WONDER, and the U.S. Department of Agriculture (USDA) has shown that household poverty is associated with increased rates of malnutrition-related deaths [54].

Further, recent data indicates that, although poverty rates among older adults declined significantly since 1975, this decline reached a plateau since approximately 2011 [55]. It is possible that the financial adversity incurred by the preceding economic depression may have adversely affected the ability of older adults to enter retirement with financial security [56]. Unlike the population overall, rates of food insecurity among older adults still had not returned to their pre-Great Recession levels, as of 2021 [53]. Many Americans rely on fixed incomes as they age [57], which can threaten their access to basic food given the ongoing retail food prices inflation since 2013 [58]. Because of the aforementioned links between economic adversity, the resulting food insecurity, and the increase in malnutrition mortality observed herein, tackling these trends will likely require stronger government-funded programs aiming to ensure food security among older adults.

The unequal distribution of modifiable risk factors, like poverty, is part of a larger problem of health disparity where certain socially disadvantaged groups systematically experience greater obstacles to health [59]. Therefore, the stratification of malnutrition mortality by certain demographics showed that while all studied groups experienced the fall and rise of the trend, they did not experience it equally. Our study found the mortality trend disproportionately affected persons ≥ 85 years of age, females, Non-Hispanic Whites, those living in the West region of the U.S., and those living in urban areas.

The steep mortality uptrend in persons ≥ 85 years of age is due to a rising average age in this subgroup [60]. As previously mentioned, aging is itself a risk factor for malnutrition [3], with several observational studies showing the likelihood of malnutrition increasing continuously with age [61–63].

As for gender, female older adults were more affected by malnutrition than their male counterparts in other studies as well [1, 64, 65]. One possible explanation for this is life expectancy, with females in the U.S. living around five years longer than males [66]. Additionally, females occupy a greater share inside nursing homes than males (67% versus 33%) [67]. Of note, some studies did not find a significant association between the female gender and malnutrition [68]. Instead, they reported that female gender is associated with functional limitation and disability, which are risk factors for malnutrition. This suggests that if the burden of functional limitation and disability which more commonly affects females can be overcome, then so can the resultant gender disparities.

Non-Hispanic Whites experienced the greatest rise in their malnutrition mortality trend. Indeed, they have multiple risk factors with reports of the highest rate of severe depression and decreased social contact compared to other races [69]. Furthermore, Whites also occupy the greatest share inside nursing homes (79% versus 15% of Blacks and 6% of Hispanics) [70]. However, it is worth noting that while Non-Hispanic Blacks had the smallest AAPC, their malnutrition mortality rate in 1999 was already high, and was consistently higher than any other included race group till the end of our study period. Previous cross-sectional analysis on community-dwelling older adults in adult day healthcare centers found that Blacks were at a greater nutritional risk than any other race group, with close to 21% reporting eating fewer than two meals per day, while around 2% of Whites reported so [69]. Moreover, a review encompassing relevant literature from 1995 to 2021 found that persistent food insecurity was more than twice as likely in African-American homes than in the general population [71].

Further, the West was the census region with the steepest rise in its malnutrition mortality trend. There are likely many contributing factors as to why this may be. Recent data from the American Community Survey shows that 11.6% of all counties in this region experienced an increase in poverty among those aged 65 and above [72]. However, it is worth noting that the Northeast, which had a lesser uptrend in mortality in our analysis, had an even greater share of counties experiencing an increase in poverty (14.8%). This suggests that other risk factors could be at play in the West.

The higher uptrends of mortality in urban (as opposed to rural) areas may at first seem perplexing in light of known difficulties in accessing healthcare in rural areas and the consequently higher rates of mortality and shorter life expectancies [73, 74]. This surprising finding may, in part, be related to differences in the degree of social and familial support between the two settings. Previous data has shown that, in urban settings, older adults report a greater degree of familial cohesion and support than in urban settings [75]. Therefore, in rural settings, older adults who suffer food insecurity may be more likely to have their nutritional needs met than in urban settings via their more robust social and familial networks. Indeed, large central metropolitan and small metropolitan areas had the sharpest uptrends in malnutrition mortality rates in our analysis. Supporting this, data from the USDA shows food insecurity rates to be highest in major metropolitan cities [76]. However, it is worth noting that both areas suffer substantial burdens of food insecurity, and differences in the prevalence thereof may not entirely explain our results, as data from the USDA shows that, on average, nonmetropolitan areas have slightly greater rates of food insecurity than metropolitan areas [76].

## Limitations

This study has several limitations. First, the determination of the underlying cause of death by clinician adjudicators carries a risk of underestimation, as malnutrition may not be readily identified in clinical practice, particularly if the individual lacks access to healthcare services and does not have a long-established relationship with a primary care physician. Therefore, the number of deaths driven by malnutrition may be underestimated. A further limitation is that ICD codes in the ICD-10/9 systems may be less suitable for high-income settings such as the U.S. This is because the clinical picture of malnutrition has evolved and is no longer necessarily associated with absolute impoverishment and deprivation but is also commonly found in adults who may, in fact, be obese but still suffer the effects of malnutrition. This likely adds to the underestimation of malnutrition-driven deaths. Third, this study did not include American Indians/Alaska Natives in the analysis stratified by race/ethnicity due to several missing data points. This is because the relatively low number of deaths leads the CDC to censor the data due to fear of individual identification. Future analyses including older individuals from these ethnicities will shed more light on malnutrition deaths in these populations. Fourth, it may have been of interest to delineate these uptrends by additional factors such as income, employment status, and the presence of health insurance. However, as CDC WONDER does not contain data on these variables, we were unable to conduct these analyses. Future analyses linking CDC WONDER data to county-level characteristics may be helpful in understanding the drivers of these deaths and the counties that may be ideal to target with interventions aimed at curbing malnutrition among older adults.

## Conclusion

The delicate balance between protective factors and risk factors drove the fall and rise of the malnutrition mortality trend between 1999 and 2020. Despite some initial decrements in malnutrition mortality among older adults in the US, the recent uptrend from 2013 to 2020 outpaced the preceding decline. The end result is that contemporary malnutrition mortality rates represent a historical high. The burden of the mortality uptrends disproportionately affected certain demographics, namely persons ≥ 85 years of age, females, Non-Hispanic Whites, those living in the West region of the U.S., and those living in urban areas. Effective interventions are needed to ensure food security and the early detection and remedy of malnutrition among older adults. Such interventions may include stronger government-funded programs and social support systems, incentives that reward the detection of malnutrition, and more cohesive patient-centered medical care for older adults.

## Data Availability

The datasets generated and analyzed during the current study are available in the CDC WONDER database, https://wonder.cdc.gov.
